# Effects of Scaffold Shape on Bone Regeneration: Tiny Shape Differences Affect the Entire System

**DOI:** 10.1021/acsnano.2c03776

**Published:** 2022-07-14

**Authors:** Koichiro Hayashi, Toshiki Yanagisawa, Ryo Kishida, Kunio Ishikawa

**Affiliations:** Department of Biomaterials, Faculty of Dental Science, Kyushu University, 3-1-1 Maidashi, Higashi-ku, Fukuoka 812-8582, Japan

**Keywords:** honeycomb, scaffold, tissue engineering, granule, regenerative medicine, bone

## Abstract

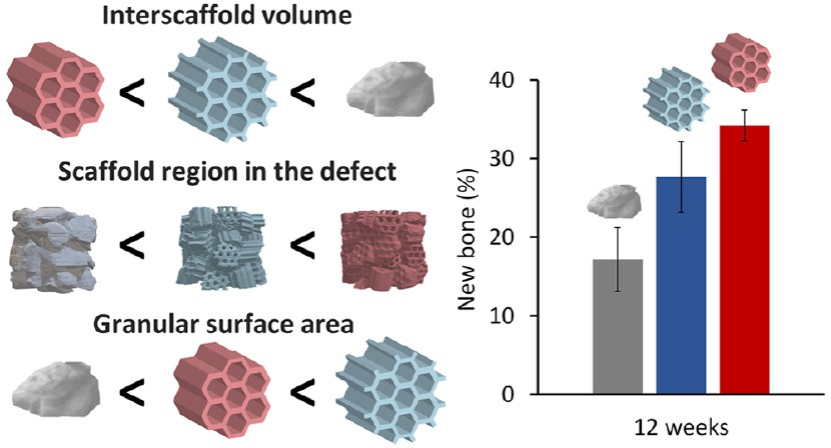

Although studies on scaffolds for tissue generation have mainly focused on the chemical composition and pore structure, the effects of scaffold shape have been overlooked. Scaffold shape determines the scaffold surface area (SA) at the single-scaffold level (i.e., microscopic effects), although it also affects the amount of interscaffold space in the tissue defect at the whole-system level (i.e., macroscopic effects). To clarify these microscopic and macroscopic effects, this study reports the osteogenesis abilities of three types of carbonate apatite granular scaffolds with different shapes, namely, irregularly shaped dense granules (DGs) and two types of honeycomb granules (HCGs) with seven hexagonal channels (∼255 μm in length between opposite sides). The HCGs possessed either 12 protuberances (∼75 μm in length) or no protuberances. Protuberances increased the SA of each granule by 3.24 mm^2^ while also widening interscaffold spaces and increasing the space percentage in the defect by ∼7.6%. Interscaffold spaces were lower in DGs than HCGs. On DGs, new bone formed only on the surface, whereas on HCGs, bone simultaneously formed on the surface and in intrascaffold channels. Interestingly, HCGs without protuberances formed approximately 30% more new bone than those with protuberances. Thus, even tiny protuberances on the scaffold surface can affect the percentage of interscaffold space, thereby exerting dominant effects on osteogenesis. Our findings demonstrate that bone regeneration can be improved by considering macroscopic shape effects beyond the microscopic effects of the scaffold.

As the global population is aging rapidly, healthy life expectancy should accompany this increase.^1,2^ To extend the healthy life expectancy, the maintenance and recovery of oral and locomotory functions are crucial, for which dental implantation and orthopedic bone regeneration are effective.^1−4^ Bone augmentation is often needed for dental implants when the alveolar bone is resorbed.^4−6^ To address this issue, granular scaffolds, rather than blockish scaffolds, are often used for bone regeneration,^4−6^ and they have been frequently used for the regeneration of intrabony defects formed by trauma and tumorectomy in orthopedic surgery.^2^ Although autologous bone grafting is currently the gold standard in bone regeneration, it can severely damage the donor site, thus providing a limited amount of harvested bone and delaying surgery.^4−11^ To counteract these drawbacks, synthetic scaffolds for bone regeneration should be developed.

The chemical composition of synthetic scaffolds for bone regeneration usually includes hydroxyapatite (HAp) and β-tricalcium phosphate (TCP).^12−16^ However, HAp is not well resorbed and remains in the body.^17^ Conversely, β-TCP resorption is excessively fast, and its function as a scaffold declines before a sufficient bone volume is reached.^18^ However, the resorption of carbonate apatite (CAp), known as a bone mineral analog, synchronizes with bone formation, and it is eventually replaced by new bone.^19−23^

Nevertheless, the chemical composition of scaffolds is only one factor affecting bone regeneration. The porosity of scaffolds is also a dominant influencing factor.^24−27^ Introducing channels (>100 μm in apertural size) into scaffolds is an effective approach to facilitate the penetration of tissues and cells into the scaffold, thereby promoting bone regeneration.^24−30^ Granular scaffolds with macropores (>100 μm in diameter) rather than channels can be fabricated using various methods.^31−38^ However, the macropores introduced into granular scaffolds by conventional approaches, such as sacrificial template and gas forming methods,^39−43^ show poor interconnectivity and irregular size and shape. In addition, not all granular scaffolds develop macropores. Although three-dimensional (3D) printing has the potential to solve these issues, it is currently unsuitable for the fabrication of granular porous scaffolds because of its low resolution and productivity.^26,44,45^ Therefore, a method of fabricating granular scaffolds with precisely controlled porous structures should be developed.

Spaces are created when bone defects are filled with granular scaffolds, and these interscaffold spaces serve as paths for the penetration of cells and tissues. However, increases in such spaces lead to a decrease in the percentage of scaffold regions in the bone defect. Therefore, interscaffold spaces and channels should be controlled to achieve effective bone regeneration. The characteristics of interscaffold spaces are dominated by the granular shape. Therefore, to clarify the effects of interscaffold spaces, the precise fabrication of granular scaffolds is essential.

Furthermore, the shape of granular scaffolds also determines the surface area (SA) of each granular scaffold. As described above, the shape of granular scaffolds often affects interscaffold spaces by altering the occupancy rate of scaffolds in the bone defect. Thus, the effect of granular scaffold shape may extend beyond the single-scaffold scale (i.e., microscopic scale) to affect the whole system (i.e., macroscopic scale). Nevertheless, these macroscopic and microscopic effects of granular scaffold shape are still not understood.

Extrusion molding is a suitable method for fabricating materials with precisely controlled porous structure and shape. We have previously fabricated CAp honeycomb scaffolds by exploiting extrusion molding.^19,20,30,46−54^ The precisely controlled porous structure promoted bone regeneration. Notably, intrascaffold channels of 230–300 μm in apertural size can prevent the penetration of fibrous tissues into the scaffold, thus providing a dominant position for osteogenesis and angiogenesis.^30,47−49^ Furthermore, among triangle-, square-, and hexagon-shaped cell geometries, the hexagonal cell has the largest area when the outer periphery lengths of these cells are equal;^55,56^ moreover, extraneous loads applied to a plane can be distributed onto the other five planes.^57^ Thus, a honeycomb structure consisting of hexagonal cells is superior in both porosity and mechanical strength. In terms of cell responses, hexagonal channels surpass triangular and square channels in terms of tissue amplification on channel faces. Therefore, honeycomb scaffolds are useful for clarifying the effects of scaffold shape and achieving effective bone regeneration.

This study presents the effects of scaffold shape on bone regeneration for three types of granular scaffolds with different shapes. Based on our previous findings, we used extrusion molding to fabricate two types of granular honeycomb scaffolds [honeycomb granules (HCGs)] with optimally sized intrascaffold channels. The HCG shapes were set by creating protuberances onto the surface while maintaining the basal honeycomb structure based on the following reasons. We hypothesized that the protuberances widen the interscaffold space and increase the SA of each granular scaffold. Therefore, we can demonstrate whether the interscaffold space or SA of each granular scaffold is the dominant parameter. Moreover, we sought to determine the influence of tiny shape differences on bone regeneration at microscopic and macroscopic scales by evaluations using the HCGs with and without protuberances. As a control scaffold, we also fabricated irregularly shaped dense granules (DGs) commonly used in dental treatments. The *in vivo* effects of HCG shape on bone regeneration were evaluated by implanting these three scaffolds into the defects of rabbit femur condyles. The findings of this study demonstrate the effects of scaffold shape at microscopic and macroscopic scales, thereby providing insights into the suitable design of materials for tissue engineering.

## Results and Discussion

### Fabrication and Characterization of Scaffolds

**Figure 1:**
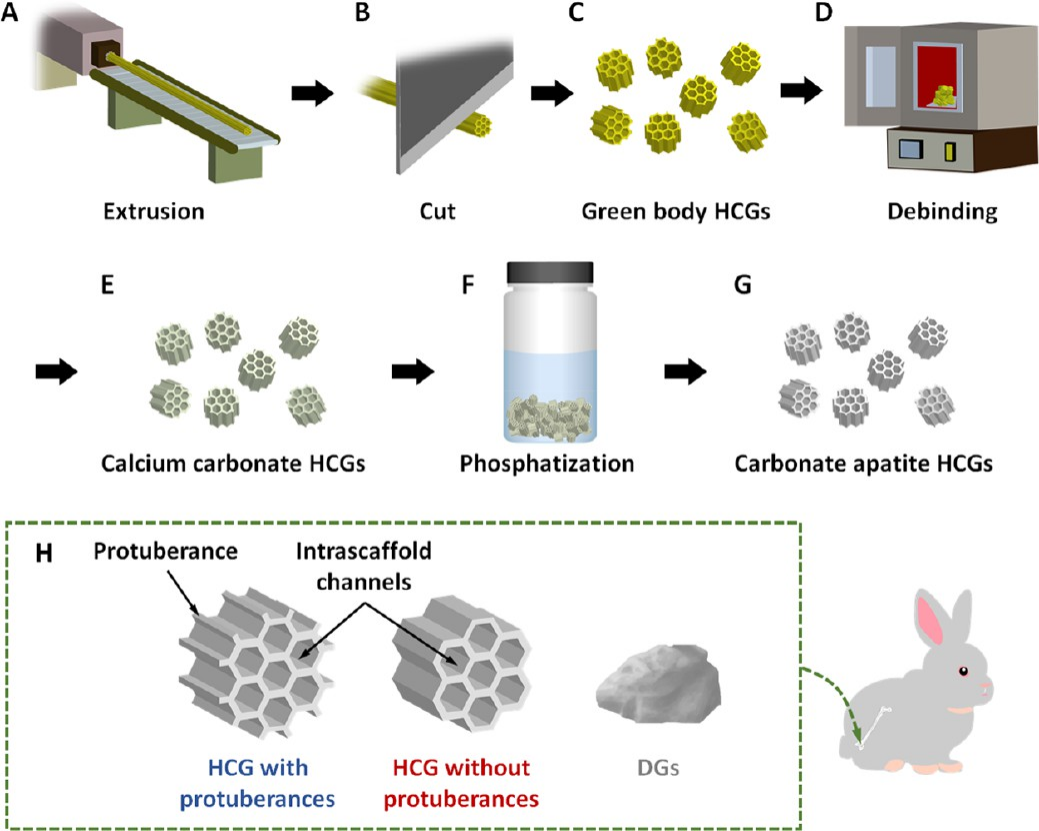
Schematic illustration of the manufacture of CAp HCGs and animal experiments: (A) extrusion process; (B) cutting of green honeycomb samples; (C) prepared green honeycomb granules (HCGs); (D) heat treatment of HCGs to obtain CaCO_3_ HCGs via debinding and sintering; (E) phosphatization of CaCO_3_ HCGs by immersion in Na_2_HPO_4_ solution; (F) preparation of CAp HCGs; (G) implantation of CAp HCGs (with or without protuberances) or DGs into a critical size bone defect in the femur condyle of rabbits.

CAp HCGs with and without protuberances were manufactured using the following procedures. First, green honeycomb sticks were prepared by extruding a mixture of calcium carbonate powder and an organic binder through a honeycomb die (Figure 1A). The honeycomb sticks were cut at 1–1.5 mm intervals using a guillotine cutter (Figure 1B), which produced green HCGs (Figure 1C). Subsequently, the organic binder was removed from the green HCGs by heating at 600 °C for 24 h (Figure 1D), and calcium carbonate HCGs were obtained (Figure 1E). Finally, calcium carbonate HCGs were subject to phosphatization in a Na_2_HPO_4_ solution at 80 °C for 7 d (Figure 1F), yielding CAp HCGs (Figure 1G). To evaluate the effects of HCG shape on the responses of *in vivo* tissues, HCGs with and without protuberances were manufactured (Figure 1H) using different HC dies corresponding to each shape based on our hypothesis that protuberances increase both the SA and interscaffold space volume of HCGs. As a control, irregular-shaped DGs, commonly used as granular scaffolds, were prepared (Figure 1H). These granules were implanted into bone defects of rabbit femur condyles (Figure 1H).

**Figure 2:**
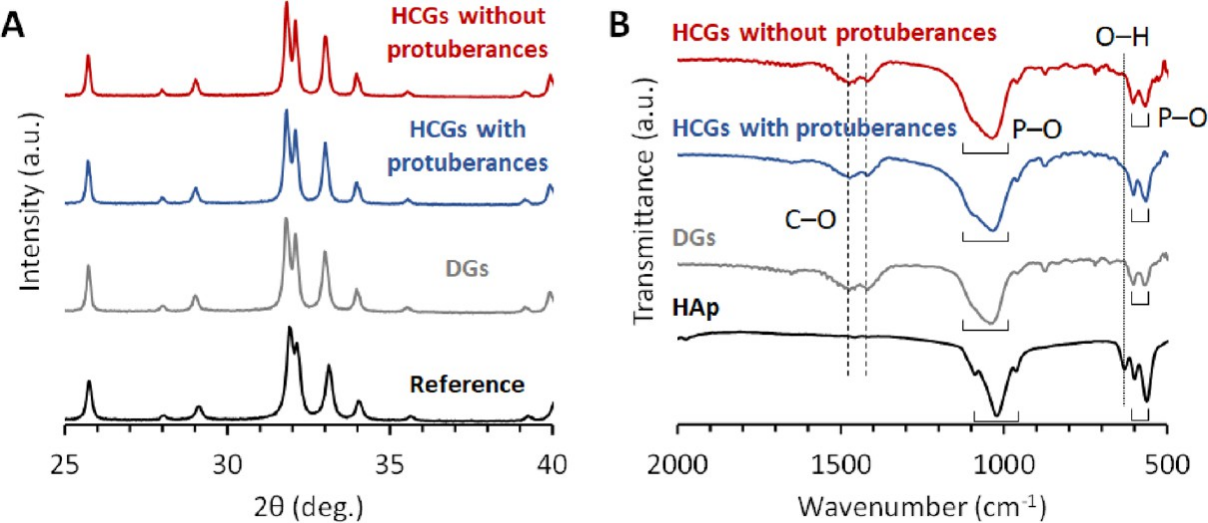
(A) XRD patterns and (B) FTIR spectra of HCGs with and without protuberances and DGs. Commercial CAp and HAp powder were used as references, respectively.

The X-ray diffraction (XRD) patterns of HCGs and DGs were consistent with that of CAp (Figure 2A). Fourier transform infrared spectroscopy (FTIR) showed phosphate absorption bands at 1136–958 cm^–1^ and 605–565 cm^–1^ in the spectra of HCGs, DGs, and HAp (Figure 2B).^58,59^ The hydroxyl absorption band was observed in the HAp spectrum (at 628 cm^–1^) but not in the spectra of HCGs and DGs.^59^ In contrast, carbonate absorption bands appeared at 1473–1409 cm^–1^ in the HCG and DG spectra but not in the spectrum of HAp.^58^ The XRD and FTIR results revealed that HCGs and DGs were composed of CAp, indicating that phosphate and hydroxyl ions in the HAp were substituted by carbonate ions.^58^

**Figure 3:**
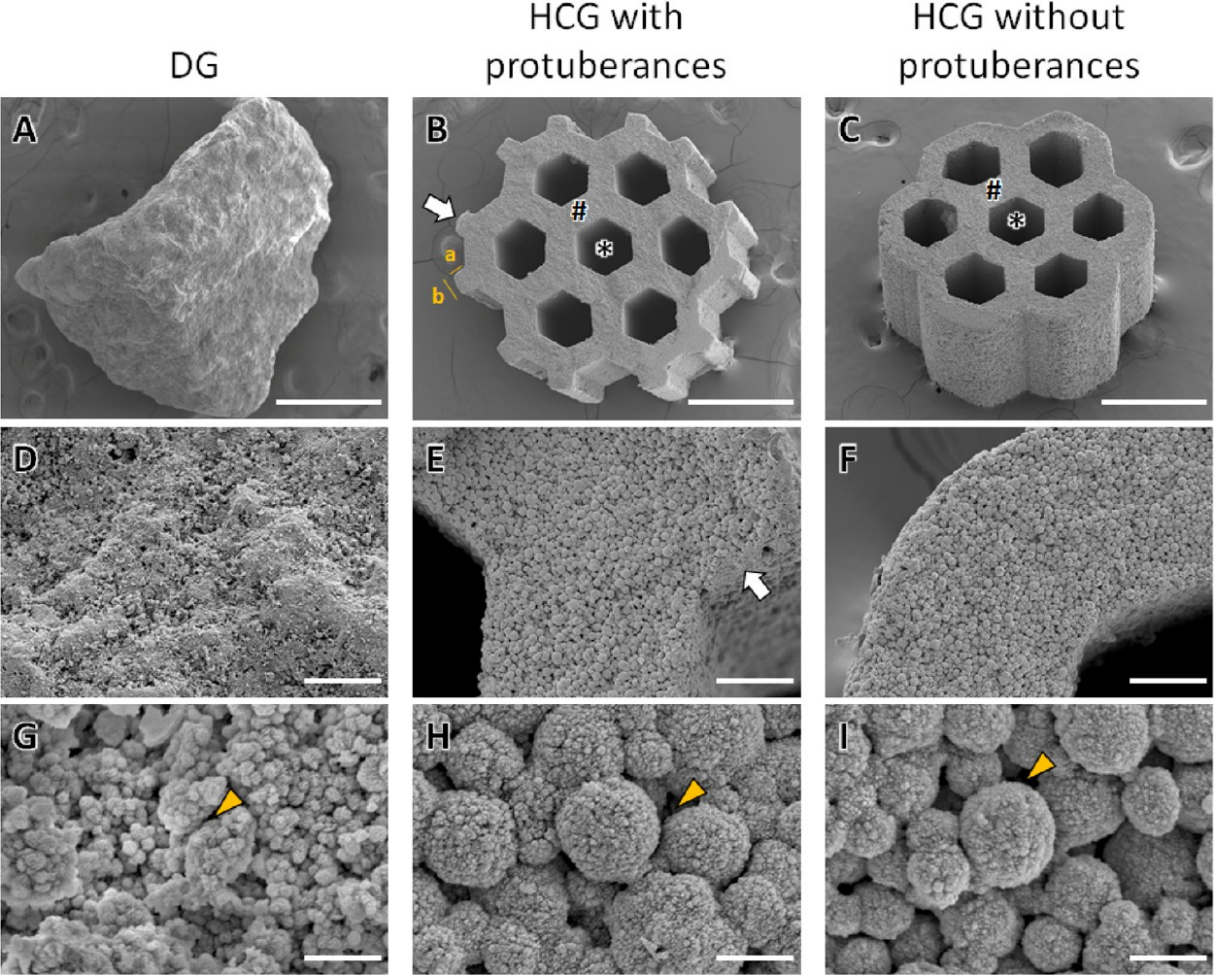
SEM images of (A) DGs and (B) HCGs with and (C) without protuberances. (D–F) High-magnification SEM images of panels A–C. (G–I) High-magnification SEM images of panels D–F. The characters “∗” and “#,” white arrows, and yellow arrowheads indicate intrascaffold channels, struts, protuberances, and micropores, respectively. Scale bars: 500 μm (A–C), 50 μm (D–F), and 5 μm (G–I).

The scanning electron microscopy (SEM) results showed that DGs presented no intrascaffold channels, and their granular size was 1.22 ± 0.27 mm (Figure 3A). Both HCGs, with and without protuberances, presented a honeycomb structure with seven hexagonal channels in the interior of scaffolds (Figure 3B,C). Although the HCGs showed similar basic shapes, there were differences associated with the presence or absence of protuberances. The lengths between the opposite sides of the intrascaffold channels in HCGs with and without protuberances were 254.1 ± 13.5 μm (Figure 3B) and 257.1 ± 12.3 μm (Figure 3C), respectively. Moreover, the strut thicknesses of HCGs with and without protuberances were 137.6 ± 8.8 μm (Figure 3B) and 137.6 ± 9.5 μm (Figure 3C), respectively. Therefore, the sizes of the intrascaffold channels and struts were equal for HCGs with or without protuberances. In contrast, the granular sizes of HCGs slightly differed according to the presence or absence of protuberances. The granular sizes of HCGs with and without protuberances were 1.30 ± 0.01 mm (Figure 3B) and 1.13 ± 0.05 mm (Figure 3C), respectively, which were consistent with the granular sizes of HCGs (Figure 3B,C). Because protuberance length a was 75.7 ± 3.2 μm (Figure 3B), the difference in granular size between HCGs with and without protuberances was consistent with the total length of two protuberances facing each other. Moreover, protuberance length b was 101.9 ± 7.5 μm (Figure 3B). Therefore, one protuberance increased the granular SA by ∼0.27 mm^2^. Because HCGs with protuberances possessed 12 protuberances (Figure 3B), the SA of an HCG with protuberances was 3.24 mm^2^ larger than that of an HCG without protuberances. The HCGs with and without protuberances and DGs consisted of spherical aggregates of crystals (Figure 3D–F) and micropores (<10 μm), between which micropores were observed (Figure 3G–I). The macroporosities of scaffolds, that is, the volume percentages of intrascaffold channels, were estimated by analyzing the μ-CT images of scaffolds using image analysis software (CT-An, Bruker, Billerica, MA, USA). The macroporosities of HCGs with and without protuberances and DGs were 36.1 ± 0.61, 38.4 ± 0.13, and 0 ± 0%, respectively. The macroporosity of HCGs with protuberances was lower than that without protuberances because the volume of HCGs with protuberances was larger than that of HCGs without protuberances. However, the volume of intrascaffold channels in the HCGs with protuberances was almost equal to that in the HCGs without protuberances. Significant differences in macroporosity were observed between HCGs with and without protuberances (*p* = 1.6 × 10^–3^). The macroporosity of DGs was zero because they possessed no channels. Thus, the macroporosity of DGs was significantly lower than those of HCGs with and without protuberances (*p* = 2.6 × 10^–8^ and 5.0 × 10^–11^, respectively).

**Figure 4:**
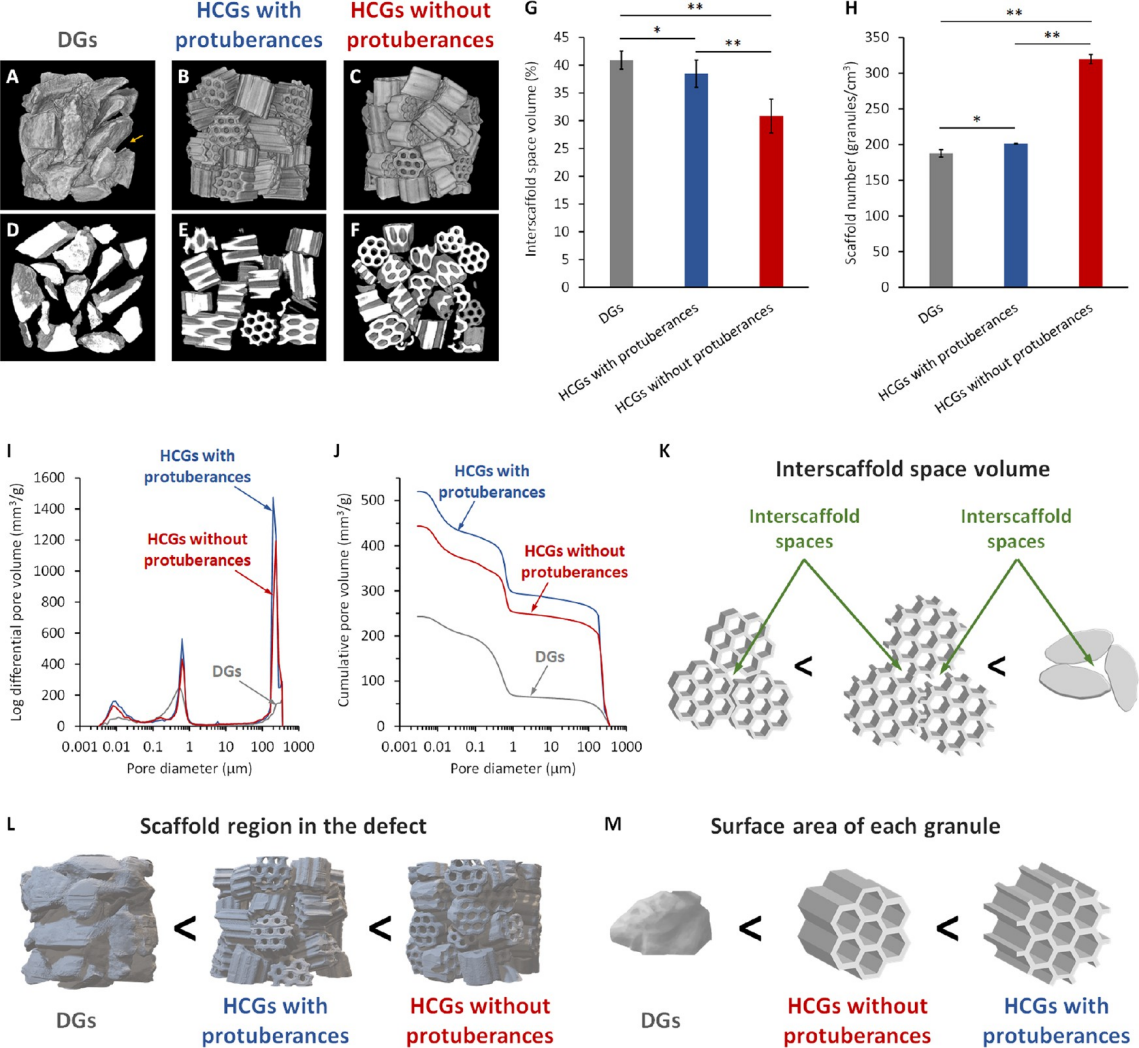
Three-dimensional reconstructed CT images of (A) DGs, (B) HCG with protuberances, and (C) HCGs without protuberances filled in a mold. The yellow arrow indicates an interscaffold space on the surface of a granular aggregate. CT images in the center regions of aggregates of (D) DGs and HCG (E) with and (F) without protuberances filled in a mold. (G) Volume percentages of interscaffold spaces acquired using CT image analysis. (H) The number of granules filled in a mold per square centimeter. Porous structure characteristics measured via mercury intrusion porosimetry: (I) pore size distribution and (J) cumulative pore volume versus pore size. Schematic illustration of the correlation between scaffold shape and (K) interscaffold space volume, (L) region functioning as cell scaffold in the system, and (M) surface area of each granular scaffold. *p < 0.05 and **p < 0.01.

The effects of granular shape on interscaffold space volume were evaluated by X-ray microcomputed tomography (CT) of granules filled into a mold that was the same size as the bone defect investigated in this study (6 mm in diameter, 5 mm in height). The 3D reconstructed images show sparsely filled DGs and large interscaffold spaces on their aggregate surface (Figure 4A). HCGs (Figure 4B,C) were more densely filled than DGs (Figure 4A). Notably, the number of HCGs without protuberances that filled into the mold (Figure 4C) was larger than that observed for HCGs with protuberances (Figure 4B). Therefore, the interscaffold spaces in the surface of the aggregates of HCGs without protuberances (Figure 4C) were smaller than those in HCGs with protuberances (Figure 4B). The CT images of the central regions of the granular aggregates clearly showed the differences in interscaffold spaces among the three scaffold types (Figure 4D–F). The HCGs without protuberances (Figure 4F) were more densely filled, and their interscaffold spaces were smaller than those of DGs (Figure 4D) and HCGs with protuberances (Figure 4E). The interscaffold space volumes were calculated based on a quantitative CT image analysis (Figure 4G). The volume percentages of interscaffold spaces in DGs and HCGs with and without protuberances were 40.9 ± 1.6%, 38.4 ± 2.4%, and 30.8 ± 3.0%, respectively (Figure 4G). The volume percentages of interscaffold spaces in HCGs without protuberances were significantly lower than those in DGs (*p* = 9.2 × 10^–5^) and HCGs with protuberances (*p* = 1.2 × 10^–3^). There was also a significant difference between DGs and HCGs with protuberances (*p* = 4.8 × 10^–2^). The number of scaffolds filled into the mold was counted as it should correlate with the interscaffold space volume and the percentage of scaffold region in the mold. The numbers of DGs and HCGs with and without protuberances per cubic centimeter were 187.7 ± 5.0, 201.2 ± 0.2, and 319.8 ± 6.5, respectively (Figure 4H). Therefore, the number of HCGs without protuberances in the mold was significantly higher than those of DGs (*p* = 4.3 × 10^–13^) and HCGs with protuberances (*p* = 1.2 × 10^–13^), and the number of HCGs with protuberances in the mold was higher than that of DGs (*p* = 2.9 × 10^–2^). In addition, the volumes of intrascaffold channels, interscaffold spaces (100–360 μm), and micropores (<10 μm) were measured with mercury intrusion porosimetry (MIP; Figures 4I and 4J). Although interscaffold spaces of diameter >360 μm were not detected by MIP because of the adopted measurement principles, the above CT image analyses compensated for this MIP drawback. The pore volumes of DGs and HCGs with and without protuberances in the pore diameter range of >100 μm were 66.6, 293.9, and 251.2 mm^3^/g, respectively (Figure 4I,J). Because DGs possessed no intrascaffold channels, the pores of DGs in the pore diameter range of >100 μm represented the interscaffold spaces. Furthermore, according to the SEM results, the intrascaffold channel sizes of HCGs with and without protuberances were equal (Figure 3A,B). Therefore, the difference in pore volume in the pore diameter range above 100 μm between the HCGs corresponded to the difference between their interscaffold space volumes. Thus, the interscaffold space volume of HCGs with protuberances was 42.7 mm^3^/g larger than that of HCGs without protuberances, in a size range of 100–360 μm.

The CT and MIP results (Figure 4A–J) demonstrated that the order of interscaffold space volume was DGs > HCGs with protuberances > HCGs without protuberances (Figure 4K), and the percentage of region functioning as cell scaffold in the mold was the opposite (HCGs without protuberances > HCGs with protuberances > DGs; Figure 4L). The order of SA of each granular scaffold was HCGs with protuberances > HCGs without protuberances > DGs (Figure 4M). Therefore, although protuberances increased the SA of each granular scaffold, they also widened the interscaffold spaces, thereby decreasing the cell scaffold region in the whole system.

**Figure 5:**
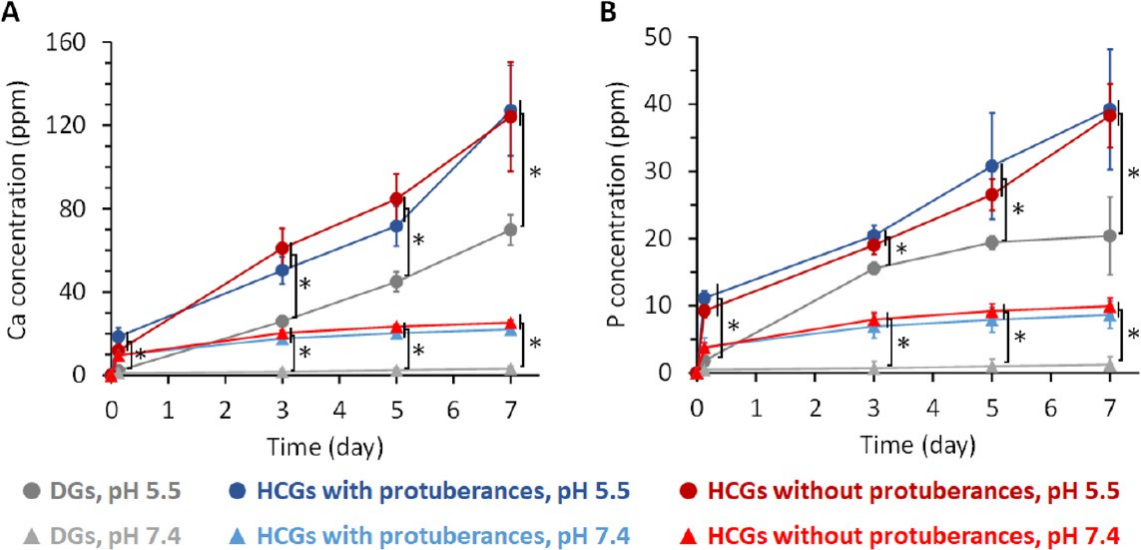
(A) Calcium and (B) phosphate ions released from HCGs and DGs in buffer solutions at pH 7.4 and 5.5. *p < 0.01.

The concentration of calcium (Figure 5A) and phosphate (Figure 5B) ions released from the HCGs and DGs in a physiological saline solution (pH 7.4) and a weak acid buffer solution (pH 5.5; resulting from the acids produced by osteoclasts) were measured by inductively coupled plasma–atomic emission spectrometry (ICP–AES). For all granules, larger amounts of calcium and phosphate ions were released at pH 7.4 than at pH 5.5 (Figure 5A,B). Furthermore, larger amounts of calcium and phosphate ions were released from HCGs than DGs at both pH values, regardless of the occurrence of protuberances (Figure 5A,B). During immersion for 7 d, the HCGs released a significantly larger amount of calcium and phosphate ions than DGs at both pH 7.4 and 5.5 (*p* < 0.05); however, no significant difference between HCGs with and without protuberances was observed because equal weights of these HCGs were used for the evaluation. These findings demonstrate that the degradation of HCGs and DGs was promoted under the weakly acidic environment produced by osteoclasts, and the intrascaffold channels of HCGs facilitated their degradation owing to the increase in granular SA.

### *In Vitro* Evaluations

**Figure 6:**
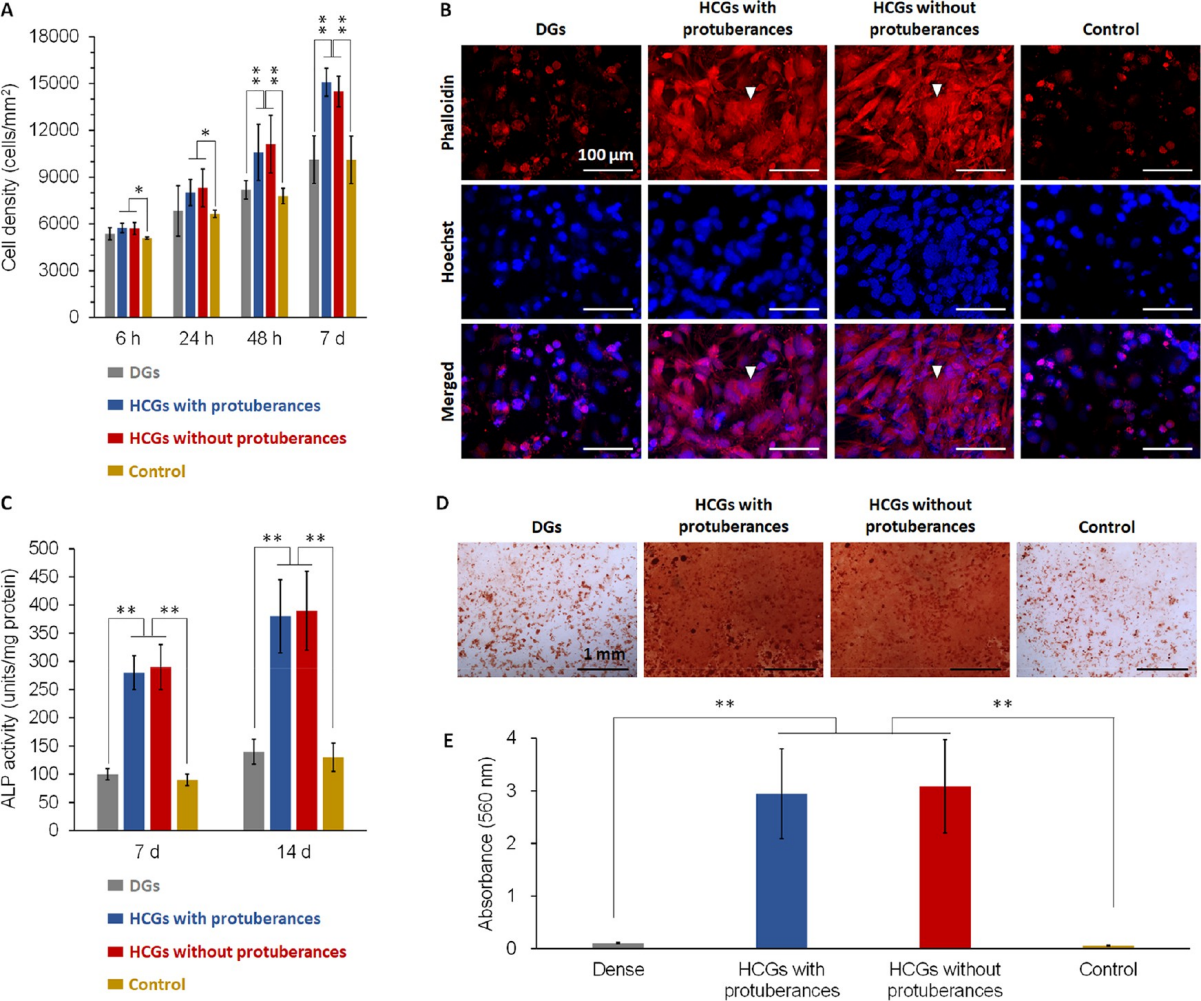
(A) Cell proliferation assays during a culture period of 7 d. (B) Fluorescent images after 7 d of culture (scale bar = 100 μm). Cellular F-actin and nuclei were stained with phalloidin and Hoechst 33258, respectively. (C) ALP activities after 7 and 14 d of culture. (D) Optical images of cultures stained with Alizarin Red S after 21 d of culture (scale bar = 1 mm). (E) Absorbance of alizarin extracts for quantification of mineral nodule formation. *p < 0.05 and **p < 0.01

Cell proliferation in the presence of DGs, HCGs with and without protuberances, and in the absence of scaffolds (control) was evaluated by assaying the cell number per square millimeter (Figure 6A). HCGs promoted cell proliferation, whereas DGs did not. The cell morphology after 7 d of culture was observed by staining cellular filamentous actin (F-actin) and nuclei (Figure 6B). The actin of cells in the HCG groups developed, whereas that in DG group did not. Furthermore, in the HCG groups, several cells were angular and dispersed, which is consistent with the common characteristics of osteogenic cells.^60^ Early osteogenic differentiation was evaluated by alkaline phosphatase (ALP) activity assay (Figure 6C). Cells in the HCG groups showed higher ALP activities than those in the DG group after 7 and 14 d of culture (Figure 6C). Mineralization was evaluated as a late marker of osteogenic differentiation by alizarin red S staining after 21 d of culture (Figure 6D). Mineralized nodules were obvious in the HCG groups (Figure 6D). The total mineralized nodule formed in each well was quantified by measuring the absorbance of alizarin extract.^61^ The amounts of mineralized nodules in the HCG groups were ∼30-fold higher than that in DG group (Figure 6E).

High concentrations of calcium and phosphate ions reportedly help promote cell proliferation, differentiation, and mineralization, whereas low concentrations of these ions do not induce these effects.^62^ Shih et al. reported that osteogenic differentiation of human mesenchymal stem cells was promoted when ∼0.3 mmol/L of calcium ions and ∼0.5 mmol/L of phosphate ions were released from scaffolds.^62^ Under physiological conditions (pH 7.4), HCGs released ∼1.0 mmol/L of calcium ions and ∼0.4 mmol/L of phosphate ions over 3 h and ∼2.0 mmol/L of calcium ions and ∼0.8 mmol/L of phosphate ions over 3 d (Figure 5A). Thus, HCGs released effective levels of calcium and phosphate ions for osteogenic differentiation. In contrast, the concentrations of calcium and phosphate ions released from DGs did not reach the effective levels even after 7 d (Figure 5A). Thus, the intrascaffold channels of HCGs increased the SA and facilitated their degradation; consequently, HCGs released effective levels of calcium and phosphate ions for osteogenic differentiation, while DGs did not. This provides an explanation for the differences in the cell proliferation, differentiation, and mineralization of HCGs and DGs.

### *In Vivo* Evaluations

**Figure 7:**
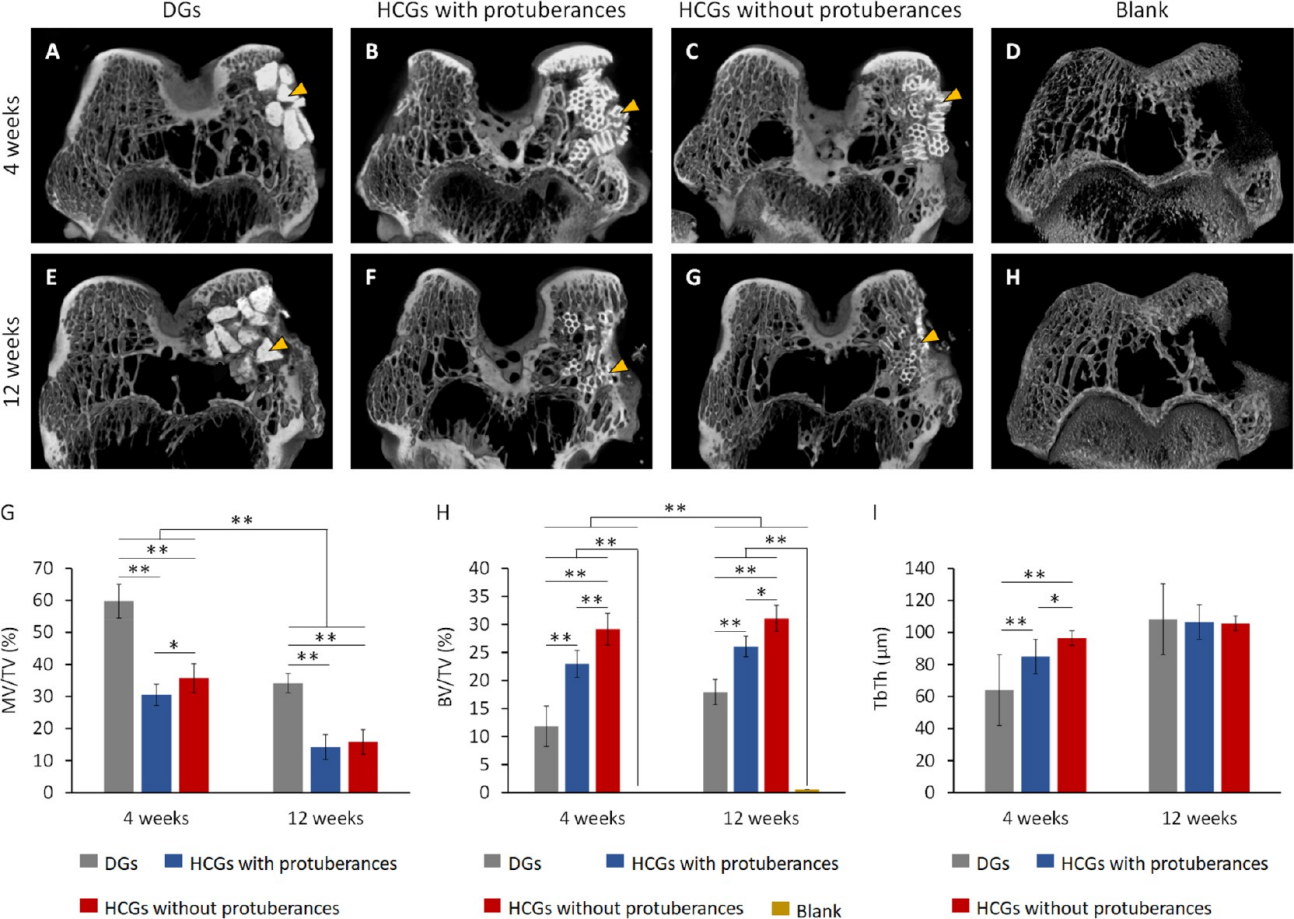
μ-CT images at (A–D) 4 and (E–H) 12 weeks after surgery. (A,E) DGs and HCGs (B,F) with and (C,G) without protuberances-groups. (D, H) Blank group (negative control). Yellow arrowheads indicate granular scaffolds. Quantitative analyses of μ-CT images: (G) MV/TV, (H) BV/TV, and (I) TbTh. *p < 0.05 and **p < 0.01. DG- and HCG-implanted groups: *n* = 6. Blank group: *n* = 4.

The X-ray μ-CT showed that radiopaque new bone was formed in the surroundings of DGs (Figure 7A) and HCGs with (Figure 7B) and without (Figure 7C) protuberances 4 weeks postimplantation (PI) in rabbit femur defects. In contrast, in the blank group, little bone was newly formed at 4 weeks after surgery (Figure 7D). At 12 weeks PI, the border between all scaffolds and new bone became vague (Figure 7E–G). In the blank group, although new bone was formed near the periosteum, massive defects remained within the bone, even 12 weeks after surgery (Figure 7H). The total volume (TV) of bone defect, material volume (MV), new bone volume (BV), and trabecular thickness (TbTh) of new bone was quantified using μ-CT images. The volume percentages of remaining materials and new bone in the bone defect were indicated as MV/TV and BV/TV, respectively. The MV/TV at 4 and 12 weeks PI were 59.8 ± 5.3 and 34.2 ± 3.0% for DGs, 30.5 ± 3.3 and 14.3 ± 3.9% for HCGs with protuberances, and 35.7 ± 4.5 and 15.9 ± 3.8% for HCGs without protuberances, respectively (Figure 7G). The BV/TV at 4 and 12 weeks PI were 11.9 ± 3.6 and 17.9 ± 2.2% for DGs, 23.0 ± 2.4 and 26.1 ± 1.8% for HCGs with protuberances, and 29.1 ± 2.8 and 31.1 ± 2.3% for HCGs without protuberances, respectively (Figure 7H). In contrast, the BV/TV was zero at 4 weeks PI and only 0.5% after 12 weeks after surgery in the blank group (Figure 7H). These findings demonstrated that a portion of the scaffolds was gradually replaced by new bone and the bone defect did not recover without these scaffolds. Furthermore, the TbTh at 4 and 12 weeks PI was 64.0 ± 4.0 and 108.3 ± 4.1 μm for DGs, 84.9 ± 6.6 and 106.5 ± 9.7 μm for HCGs with protuberances, and 96.5 ± 7.8 and 105.7 ± 8.8 μm for HCGs without protuberances, respectively (Figure 7I). For all of these scaffolds, the TbTh gradually increased and reached the same level as the original trabeculae at 12 weeks PI. Notably, the HCGs without protuberances formed a larger volume of new bone with thicker trabeculae than the HCGs with protuberance and DGs.

**Figure 8:**
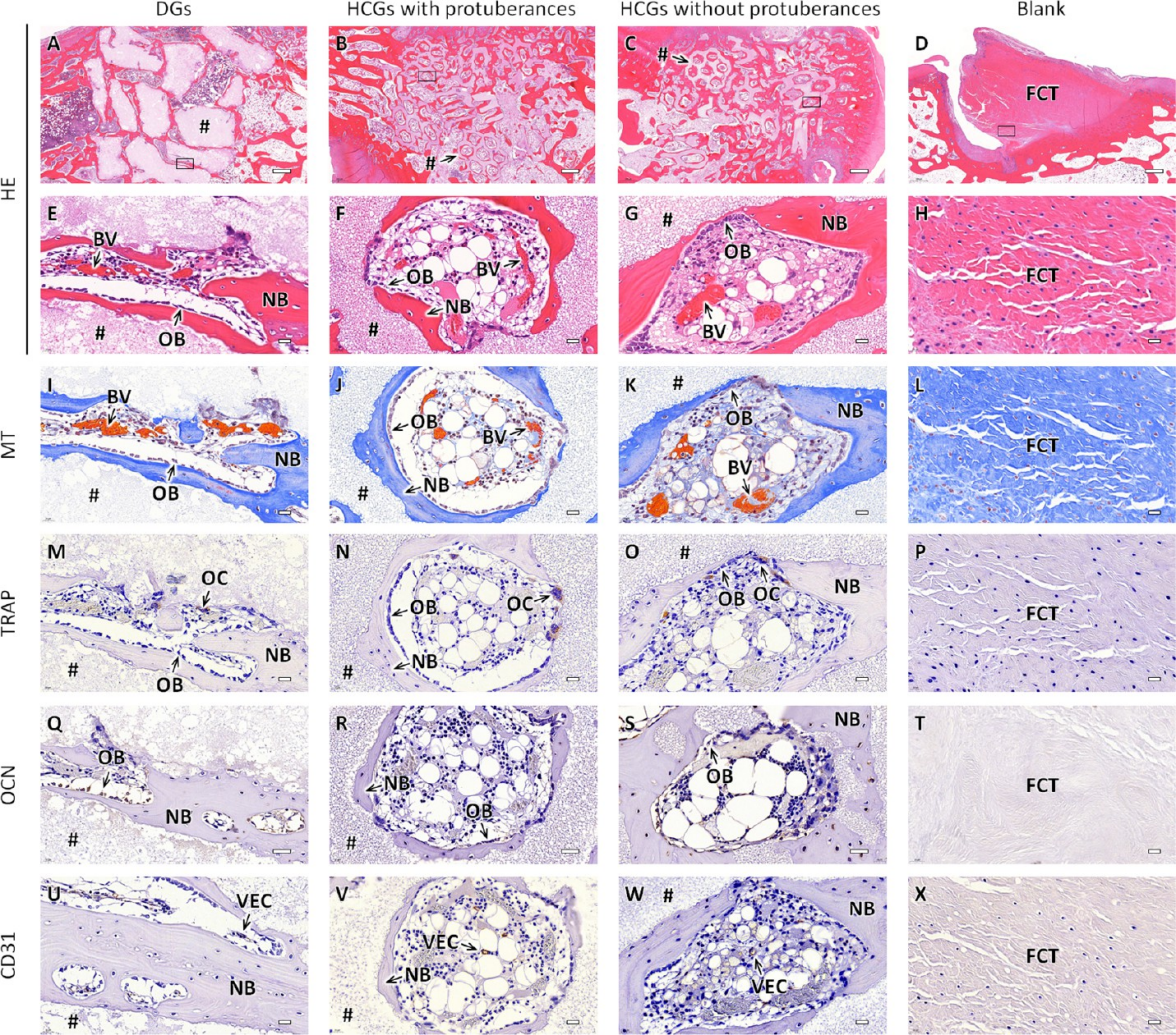
Histological sections of the DG- and HCG implanted groups with and without protuberances and blank group at 4 weeks after surgery. (A–H) HE, (G–L) MT, (M–R) TRAP, (M–R) OCN, and (U–X) CD31-stained sections. Panels E–X showed magnified images of the regions corresponding to the regions enclosed by squares in panels A–D. NB, BV, OB, OC, VEC, FCT, and “#” indicate new bone, blood vessel, osteoblast, osteoclast, vascular endothelial cell, fibroconnective tissue, and remaining scaffold, respectively. Scale bars: 500 μm (A–D) and 20 μm (E–X).

The responses of cells and tissues and the resorption of scaffolds were analyzed in detail by hematoxylin-eosin (HE), Masson trichrome (MT), tartrate-resistant acid phosphatase (TRAP), osteocalcin (OCN), and CD31 staining of histological sections. The HE-stained sections at 4 weeks PI indicated that in the DG-implanted group, new bone was formed only on the scaffold surface (Figure 8A), whereas in the HCG-implanted groups, new bone was formed both on the scaffold surface and the interior of intrascaffold channels irrespective of the presence of protuberances (Figure 8B,C). In the blank group, bone defects were filled with fibroconnective tissues (Figure 8D). High-magnification images of the DGs-implanted group showed that osteoblasts resided on the bone formed on the scaffold surface, and blood vessels were formed in interscaffold spaces (Figure 8E). In the HCGs-implanted groups, osteoblasts resided on new bone, and blood vessels were formed in both intrascaffold channels and interscaffold spaces (Figure 8F,G). In the blank group, fibroconnective tissues filling the bone defect were dense (Figure 8H). In MT-stained sections (Figure 8I–L), collagen fibers in bones and mature bones were stained blue and red, respectively.^63^ In the DG- and HCG-implanted groups, new bone was mainly stained blue and partly stained red (Figure 8I–K). In the blank group, fibroconnective tissues filling the bone defect were stained blue, indicating that the tissues were collagenous (Figure 8L). In the TRAP-stained sections, osteoclasts were stained brown (Figure 8M–O). TRAP-positive cells resided on the scaffold surfaces in the DG- and HCG-implanted groups (Figure 8M–O), whereas TRAP-positive cells were not present in the blank group (Figure 8L). In the OCN-stained sections of the DG- and HCG-implanted groups, OCN-positive cells, such as osteoblasts, were stained brown and lined the new bone tissue (Figure 8Q–S). In contrast, in the blank group, OCN-positive cells were not observed (Figure 8T). In the DG- and HCGs-implanted groups, vascular endothelial cells (VECs) in the CD31-stained sections were stained brown, and lined blood vessels formed in the interscaffold spaces and intrascaffold channels (Figure 8U–W).

**Figure 9:**
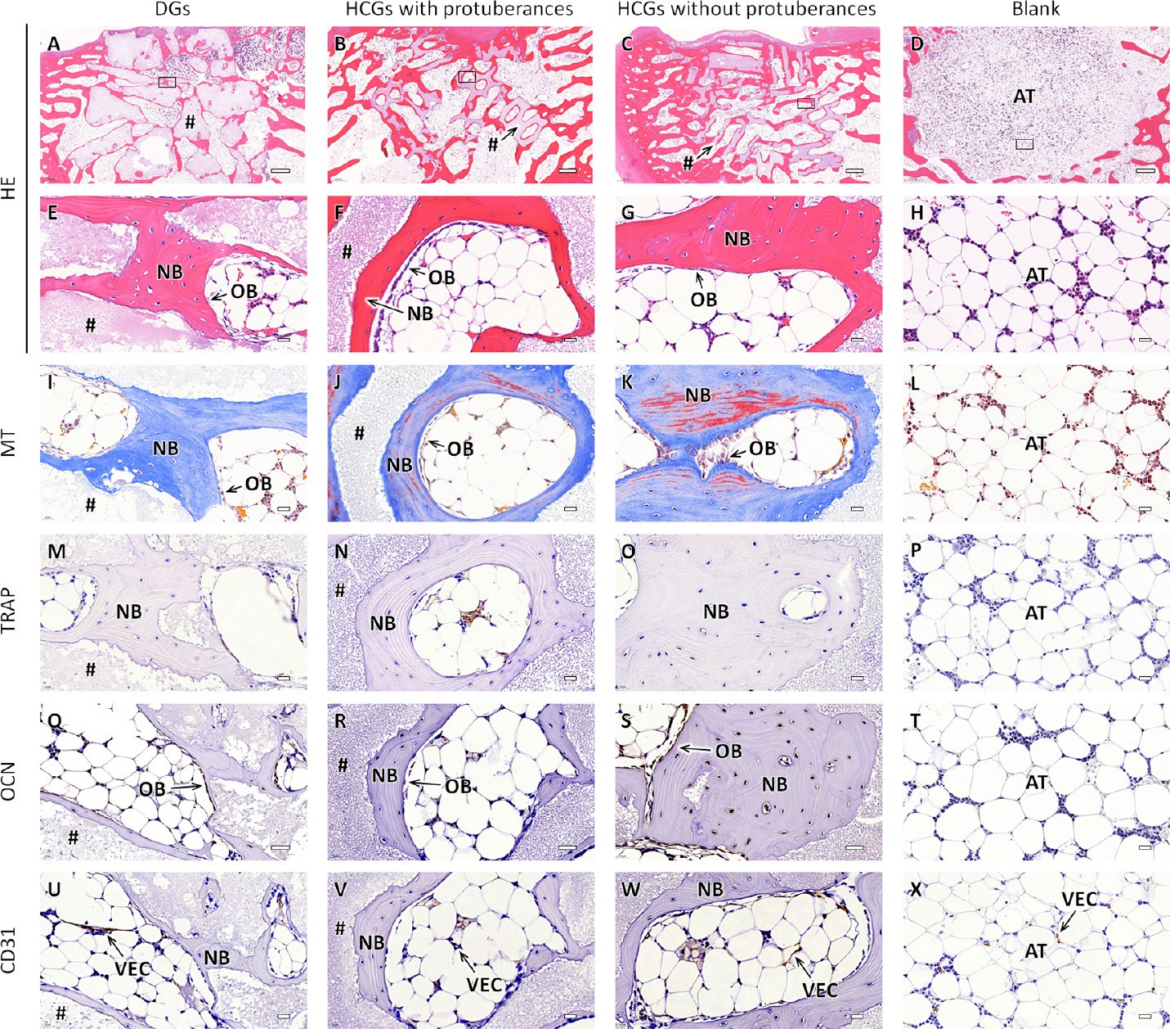
Histological sections of the DG- and HCG implanted groups with and without protuberances and blank group at 12 weeks after surgery. (A–H) HE, (G–L) MT, (M–R) TRAP, (M–R) OCN, and (U–X) CD31-stained sections. Panels E–X are magnified images of the regions corresponding to the regions enclosed by squares in the panels A–D. NB, BV, OB, OC, VEC, AT, and “#” indicate new bone, blood vessel, osteoblast, osteoclast, vascular endothelial cell, adipose tissue, and remaining scaffold, respectively. Scale bars: 500 μm (A–D) and 20 μm (E–X).

At 12 weeks PI, scaffolds were resorbed in the DG- and HCG-implanted groups compared to the results at 4 weeks PI (Figure 9A–C). In contrast, in the blank group, the bone defect was filled with adipose tissue (Figure 9D). High-magnification images of the DG- and HCG-implanted groups showed that the regions where scaffolds were resorbed were filled with new bone, that is, the scaffolds were replaced with new bone (Figures 9E–G). In contrast, in the blank group, adipose tissues dominated the bone defect (Figure 9H). The MT-stained sections in the DGs-implanted group showed a large portion of blue-stained bone tissue (Figure 9I). Conversely, in the HCGs-implanted groups, bone was extensively stained in red (Figure 9J,K). Therefore, bone tissues in the HCGs-implanted groups (Figure 9J,K) were more mature than those in the DGs-implanted group (Figure 9I). Furthermore, the bone maturity in the HCGs-implanted groups increased throughout 4–12 weeks PI (Figures 8J,K, and 9J,K), whereas the bone maturity in the DGs-implanted group did not change substantially (Figures 8I and 9I). In the MT-stained sections of the blank group, collagenous tissue disappeared (Figure 9L). In the TRAP-stained sections, the numbers of osteoclasts in the DG- and HCG-implanted groups dramatically decreased at 4–12 weeks PI (Figure 9M–O). In the blank group, TRAP-positive cells were still absent (Figure 9P). In the OCN-stained sections of the DG- and HCG-implanted groups, similar to the findings at 4 weeks PI, OCN-positive cells resided on the new bone surface (Figure 9Q–S). In the blank group, no OCN-positive cells were present in the bone defect (Figure 9T). In the CD31-stained sections, VECs remained in the interscaffold spaces and intrascaffold channels (Figure 9U–W). In the blank group, CD31-positive cells were observed between adipose cells (Figure 9X).

**Figure 10:**
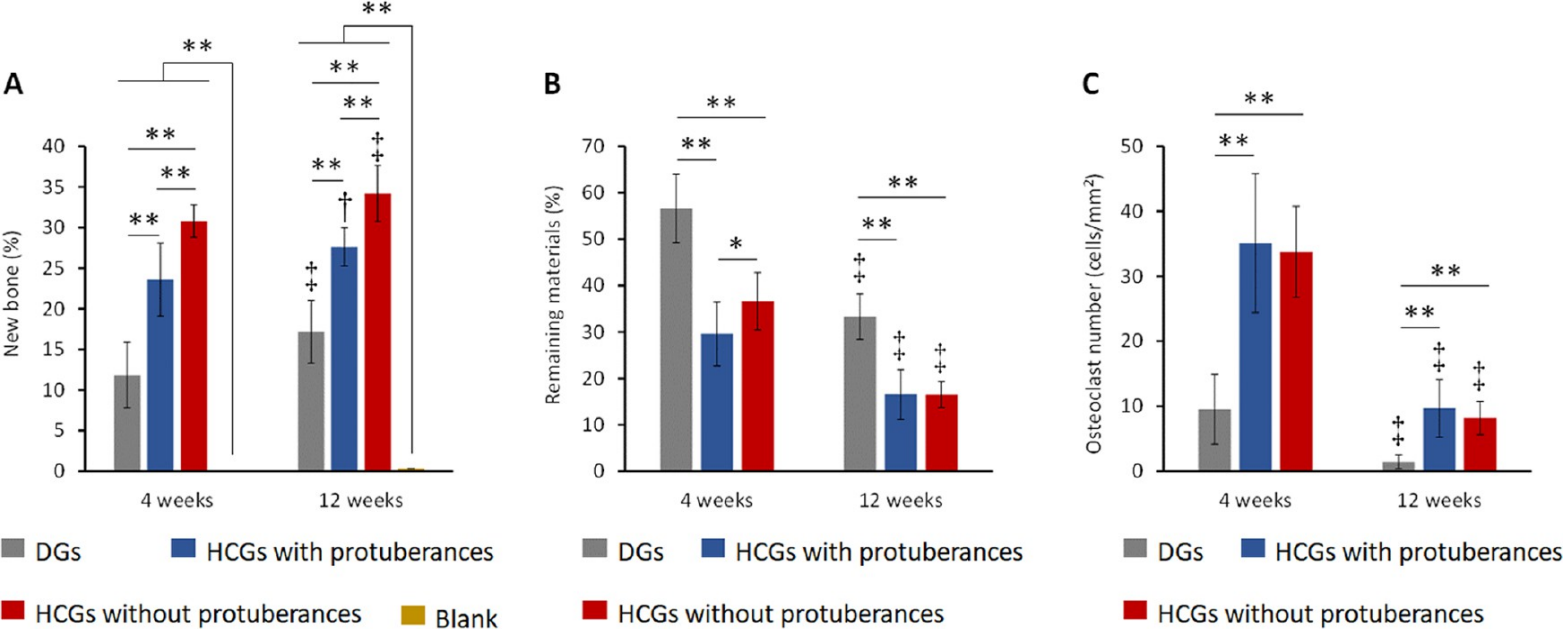
Percentages of (a) new bone and (b) remaining materials and (c) number of osteoclasts on granular scaffolds. *p < 0.05, **p < 0.01. ^†^Significant difference compared with the results at 4 weeks after surgery. DG- and HCG-implanted groups: *n* = 6. Blank group: *n* = 4.

To quantitatively analyze the effects of granular shape on biological responses, the area percentages of new bone and remaining materials and the number of osteoclasts of histological sections were estimated. Both at 4- and 12-weeks PI, the percentages of new bone area (NBA) in the HCGs-implanted groups were higher than those in the DGs-implanted group (Figure 10A), demonstrating that the intrascaffold channels contributed to effective bone ingrowth into the scaffold. The group implanted with HCGs with protuberances showed a lower NBA percentage than that implanted with HCGs without protuberances (Figure 10A). Thus, the presence of protuberances adversely affected new bone formation. This fundamental reason was because the presence of the protuberance decreased the number of scaffolds in the bone defect (Figure 4H), which caused a reduction in the total SA of scaffolds in the defect (Figure 4L). That is, the region for the attachment, proliferation, and differentiation of osteogenesis-related cells was reduced. Owing to the above effects of intrascaffold channels and protuberances, the HCGs without protuberances had the highest bone formation rate. In the DG- and HCG-implanted groups, the NBA percentages increased during the observation period (Figure 10A). At 4- and 12-weeks PI, the percentages of remaining material area (RMA) in the DGs-implanted group were higher than those in the HCGs-implanted groups (Figure 10B), explained by the DGs’ lack of intrascaffold channels. At 4 weeks PI, the RMA percentage in the group implanted with HCGs without protuberances was higher than that in the group implanted with HCGs with protuberances (Figure 10B). This difference in RMA percentage correlated with the *in vitro* granule number in the mold (Figure 4L). In the DG- and HCG-implanted groups, the RMA percentages decreased between 4- and 12-weeks PI (Figure 10B), demonstrating that all scaffolds were gradually resorbed. Furthermore, both at 4- and 12-weeks PI, the osteoclast numbers in the DGs-implanted group were smaller than those in the HCGs-implanted groups (Figure 10C). This occurred because the DGs did not possess intrascaffold channels (Figure 3A), and they presented a smaller scaffold area for osteoclasts than HCGs (Figure 4L). There was no significant difference between the osteoclast number per square millimeter of HCGs with and without protuberances (Figure 10C). In the DG- and HCG-implanted groups, the osteoclast numbers decreased from 4 to 12 weeks PI (Figure 10C). The above *in vivo* results demonstrate that all scaffolds were gradually resorbed by osteoclasts and replaced by new bone. As the replacement of scaffolds by new bone progressed, the number of osteoclasts decreased. In the blank group, no new bone formed at 4 weeks after surgery and the NBA percentage was only 0.3%, even at 12 weeks after surgery (Figure 9A). Thus, the bone defect was not spontaneously resolved without the scaffolds.

To improve the osteogenesis ability of scaffolds, previous studies mainly focused on the effects of raw material composition, substituted or doped ions, pore characteristics, and incorporation of growth factors, polysaccharides, stem cells, and extracellular vesicles.^16,64−71^ However, the effects of scaffold shape on bone regeneration remained unclear. To date, the shape of granular scaffolds has not been considered; granular scaffolds are often manufactured by smashing the bulk material, producing irregular-shaped granules. Here, we investigated the effects of granular shapes, which were precisely controlled. The main discovery was that scaffold shape, even with the presence of tiny protuberances, profoundly affects the interscaffold space percentage in the bone defect, ultimately yielding greater effects than the SA of each scaffold. Thus, the shape difference at the single scaffold scale (i.e., microscopic scale) affects the cell scaffold area in the whole system (i.e., macroscopic scale).

In the present study, the protuberance length was set to half the length of the strut for the initial trial study performed to demonstrate the effects of small differences in scaffold shape on bone regeneration. When the protuberance is longer, steric hindrance may be greater, which may widen the interscaffold space and decrease the number of scaffolds in the bone defect. As a result, new bone formation may decrease. In contrast, when the protuberance is shorter, the effect of the SA of each scaffold might surpass that of the scaffold number in the bone defect, resulting in a higher rate of new bone formation. The scaffold shape in this study is not optimal for every bone defect because the ideal shape depends on the shape and size of the bone defect. Nevertheless, the findings in this study are universally applicable because scaffold shape should be designed by considering macroscopic scale effects wielded by microscopic differences. Thus, the bone regeneration ability of scaffolds can be further enhanced by considering scaffold shape as a key parameter in scaffold design. The incorporation of computational science may help design scaffold shapes depending on the characteristics of the tissue defect.

## Conclusions

In this study, we fabricated HCGs with and without protuberances and DGs. The presence of protuberances decreased the cell scaffold region of HCGs in the system but increased the SA of each HCG. The total SA of DGs in the system and the SA of each DG were lower than those of HCGs because of their irregular shape and absence of intrascaffold channels, respectively. Owing to the intrascaffold channels, HCGs formed larger amounts of new bone than DGs. In addition, HCGs without protuberances formed larger amounts of new bone than HCGs with protuberances. Therefore, the number of cell scaffolds in the whole system (macroscopic effects of scaffold shape) was the driving factor for bone regeneration rather than the SA of each granular scaffold (i.e., microscopic effects of scaffold shape).

## Methods

### Fabrication of Granular Scaffolds

HCGs were fabricated using a method modified from our previous report,^49^ as illustrated in Figure 1. First, honeycomb green sticks were manufactured by extrusion molding using CaCO_3_ powder (Sakai Chemical Industry, Osaka, Japan) and an acryl resin-based binder (Nagamine Manufacturing, Kagawa, Japan) in an extruder (Lab Plastmill, Toyo Seiki Seisaku-sho Ltd., Tokyo, Japan). The green honeycomb sticks were extruded on a conveyor belt (Figure 1A) and then cut with a guillotine cutter into 1–1.5 mm-long granules (Figure 1B) to produce green HCGs (Figure 1C). Subsequently, green HCGs were heated at 600 °C for 24 h (Figure 1D) to remove the organic binder, yielding CaCO_3_ HCGs (Figure 1E). Finally, to convert the chemical composition of HCGs from CaCO_3_ to CAp through dissolution–precipitation reactions, CaCO_3_ HCGs were immersed in a 1 mol/L Na_2_HPO_4_ solution (Fujifilm Wako Pure Chemical, Osaka, Japan) and phosphatized at 80 °C for 7 d (Figure 1F). The obtained CAp HCGs (Figure 1G) were washed thoroughly with distilled water.

### Physicochemical Properties

The crystal phases of HCGs and DGs were determined using XRD (D8 Advance, Bruker AXS GmbH, Karlsruhe, Germany). Functional groups in the HCGs and DGs were determined using FTIR spectrophotometry (FT-IR-6200, JASCO, Tokyo, Japan). Commercial CAp granules (GC Corporation, Tokyo, Japan) and HAp powder (Taihei Chemical Industrial, Osaka, Japan) were used as references. Microstructures and shapes of HCGs and DGs were observed by SEM (S3400N, Hitachi High-Technologies, Tokyo, Japan). The porous characteristics of HCGs and DGs were measured by MIP (AutoPore 9420, Shimadzu Corporation, Kyoto, Japan). The macroporosities of the HCGs and DGs were calculated from the following equation (*n* = 20):
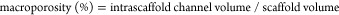
1

Intrascaffold channel volume and scaffold volume were quantified using quantitative 3D analysis software for CT images (CT-An, Bruker Corporation). To estimate these volumes, the whole scaffold was set as the region of interest (ROI). The channels and struts were easily distinguished (Figure S1) and used to calculate the macroporosity.

### Percentage and Volume of Interscaffold Space and Granular Number

The percentage and volume of interscaffold space were analyzed by an X-ray μ-CT (SkyScan, Bruker Corporation) analysis of granular scaffolds filled into a cylindrical Styrofoam mold (6 mm in diameter and 5 mm in height). CT analyses were conducted using quantitative 3D analysis software for CT images (CT-An, Bruker Corporation). To determine the granular scaffold number per cubic centimeter, the granular scaffolds were filled into the mold, and they were taken out from the mold. The number of scaffolds taken was counted. These counting procedures were repeated five times, and the average and standard deviation were calculated.

### Release of Ions from Granular Scaffolds into Buffer Solutions

HCGs and DGs (50 mg) were immersed in a 0.05 mol/L tris(hydroxymethyl)aminomethane–HCl buffer solution (20 mL) at pH 7.4 (corresponding to physiological pH) and 0.08 mol/L acetic acid–sodium acetate solution (20 mL) at pH 5.5 (corresponding to a weakly acidic environment because of osteoclast-produced acids). After immersion for 3 h, 1 d , 3 d, and 7 d, supernatant samples were collected. The concentrations of calcium and phosphate ions released from the HCGs and DGs in the supernatants were measured by inductively coupled plasma–optical emission spectrometry (ICP–OES, Optima 7300 DV, PerkinElmer, MA, USA).

### *In Vitro* Cell Proliferation, ALP Activity, and Mineralization Assays

Human umbilical cord mesenchymal stem cells (JCRB Cell Bank, Osaka, Japan) were used to evaluate cell proliferation, ALP activity, and mineralization in the presence of HCGs and DGs. Cells were seeded in a 24-well plate at 1.0 × 10^5^ cells/well and cultured in a medium (Plusoid-M, GlycoTechnica, Kanagawa, Japan) for 24 h at 37 °C in a humidified atmosphere containing 5% CO_2_. Subsequently, HCGs or DGs filled in cell culture inserts containing a 0.4-μm transparent polyethylene terephthalate membrane (Corning, NY, USA) were set at each well. After 6, 24, 48 h, and 7 d of culture, the cell number per well (*n* = 6) was assayed using a water-soluble tetrazolium (WST) salt (WST-8 assay kit, DOJINDO, Kumamoto, Japan). The cell proliferation was evaluated by comparing the cell number per well cultured without materials, that is, the control group.

For cytoskeleton observations, after 7 d of culture, cells were fixed with a formalin solution (FUJIFILM Wako Pure Chemical) for 30 min. The fixed cells were permeabilized with 1% polyethylene glycol mono-4-octylphenyl ether (Triton X-100, Nacalai Tesque, Inc., Kyoto, Japan) for 5 min at room temperature. Then, the F-actin cytoskeleton and nucleus were stained with fluorescent-labeled phalloidin (Acti-stain 555 phalloidin, Cytoskeleton, Inc., CO, USA) and Hoechst 33258 (DOJINDO). The stained cells were observed using a fluorescent microscope (BZ-X, Keyence, Osaka, Japan).

ALP activities were evaluated after 7 and 14 d of culture by the following procedures. Cells were washed three times with sterile PBS and lysed with 1% Triton X-100 (Nacalai Tesque, Inc.) for 30 min. The ALP activity and the total protein concentrations of the cells were measured according to the instructions of the ALP assay kit (LabAssay ALP, FUJIFILM Wako Pure Chemical) and protein assay kit (Protein Assay Rapid Kit wako II, FUJIFILM Wako Pure Chemical), respectively. The absorbance at wavelengths of 405 and 600 nm corresponding to the ALP activity and total protein concentration, respectively, were measured using a microplate reader (Multiskan FC, Thermo Scientific, MA, USA).

Mineralization was evaluated after 21 d of culture by the following procedures. Cells were fixed with a formalin solution (FUJIFILM Wako Pure Chemical) and stained with an Alizarin Red S staining kit (Cosmo Bio, Tokyo, Japan). The cells were washed six times with distilled water, and optical images were taken with a microscope (BZ-X, Keyence). The total mineralized nodules formed in each well were quantified according to the method of Lee et al.^61^ Alizarin Red S was extracted from the stained sites by adding 10% (w/v) cetylpyridinium chloride (Tokyo Chemical Industry, Tokyo, Japan) buffer in 10 mM Na_2_HPO_4_ (FUJIFILM Wako Pure Chemical) and maintaining the samples overnight at 37 °C. Then, 200 μL aliquots were transferred to a 96-well plate, and the absorbance at 560 nm was measured using a microplate reader (Multiskan FC). The absorbance difference between the sample and blank groups was also calculated.

### Ethics Statement

All animal experiments were conducted according to the ethical policies and procedures approved by the Animal Care and Use Committee of Kyushu University, Japan (Approval No. A30-237-0; issued August 1, 2018).

### Animals

Japanese white rabbits (18 weeks of age, 3.0–3.5 kg of body weight) were purchased from Japan SLC (Shizuoka, Japan). Rabbits were single-housed in cages and maintained on a standard diet with an adequate amount of water at the Center of Biomedical Research, Research Center for Human Disease Modeling, Graduate School of Medical Sciences, Kyushu University. Eighteen rabbits (36 legs) were used for the HCG and DG groups (*n* = 6 per group), and four rabbits were used for the negative control group (*n* = 4). A total of twenty-two rabbits were used.

### Surgical Procedure

To prepare a critical-size bone defect, which is a wound that will not heal spontaneously despite surgical stabilization and requires further surgical intervention, a sufficiently large rabbit femur condyle was used to prepare the critical-size bone defects.^72^ Rabbits were subjected to an intramuscular anesthesia injection of xylazine (5.0 mg/kg) and ketamine (30 mg/kg). The femur area of the rabbits was shaved on both sides. The femoral skin was disinfected with 10% w/v povidone–iodine (Meiji Seika Pharma, Tokyo, Japan). The femur condyle was exposed by making an incision in the femoral skin (approximately 2 cm in length) using a scalpel. The periosteum was separated from the bone using a raspatory. HCGs and DGs were separately implanted into the critical size defects (6 mm diameter, 3 mm depth) produced in the femur condyles of both legs. The periosteum and subsequently the incised skin were sutured. Finally, the surgical site was disinfected with 10% w/v povidone–iodine and, subsequently, a gentamicin sulfate solution (Gentacin, Takata Pharmaceutical, Saitama, Japan) was intraperitoneally injected to prevent infection.

### Radiographic and Histological Analyses

Four and 12 weeks after the implantation of HCGs and DGs, rabbit femurs (*n* = 6 per group) were collected and immersed in a formalin solution to fix them. The μ-CT images of the scaffolds-implanted regions were obtained by μ-CT scanning (SkyScan, Bruker Corporation). For the μ-CT analyses, the whole bone defect area was set as the ROI. An example ROI set in a slice image is shown in Figure S2A in the Supporting Information. ROIs were similarly set in all images. The bone ROI was clearly extracted (Figure S2B). Similarly, the material ROI was easily extracted. The BV and MV relative to the TV of the bone defect and the TbTh were quantified using quantitative 3D analysis software for CT images (CT-An, Bruker Corporation). The volume percentages of new bone and remaining scaffold in the bone defect were indicated as BV/TV and MV/TV, respectively.

After the μ-CT scanning, the specimens were decalcified, paraffin-embedded, and sliced into sections, which were treated with HE, TRAP, OCN, CD31, and MT staining. The histological images of the HE-, MT-, OCN-, CD31-, and TRAP-stained tissue sections were obtained using a microscope (BZ-X, Keyence). The share of new bone and remaining material areas and the number of osteoclasts were estimated using the BZ-X digital analysis software for the stained sections.

### Statistical Analysis

Statistical analyses were conducted using KaleidaGraph version 4.5 (Synergy Software, PA, USA). All data are presented as the mean ± standard deviation, and *p*-values < 0.05 were considered statistically significant. Differences were determined based on a one-way analysis of variance (ANOVA) and for significant differences, the post hoc Tukey-Kramer Multiple Comparison Test was conducted.

## Supporting Information Available

Figure S1 and Figure S2 show the methods for quantitative analyses of the macroporosity of granular scaffolds and in vivo results using μ-CT images, respectively (PDF)

## Abbreviations

HAp: hydroxyapatite
TCP: tricalcium phosphate
CAp: carbonate apatite
3D: three-dimensional
SA: surface area
HCGs: honeycomb granules
DGs: dense granules
XRD: X-ray diffraction
FTIR: Fourier transform infrared
SEM: scanning electron microscopy
μ-CT: microcomputed tomography
MIP: mercury intrusion porosimetry
ICP–AES: inductively coupled plasma–atomic emission spectrometry
ALP: alkaline phosphatase
TV: total volume
MV: material volume
BV: bone volume
TbTh: trabecular thickness
HE: hematoxylin-eosin
MT: Masson trichrome
TRAP: tartrate-resistant acid phosphatase
OCN: osteocalcin
VECs: vascular endothelial cells
NBA: new bone area
RMA: remaining material area
PI: postimplantation
WST: water-soluble tetrazolium
