# Supplementary material for: Effects of Scaffold Shape on Bone Regeneration: Tiny Shape Differences Affect the Entire System
Source: ACS Nano. 2022 Jul 14;16(8):11755–68. doi: 10.1021/acsnano.2c03776 (PMC9413413; doi:10.1021/acsnano.2c03776)
Supplement: Supplementary file 1 — nn2c03776_si_001.pdf [file nn2c03776_si_001.pdf]

Supporting Information

Effects of Scaffold Shape on Bone Regeneration: Tiny Shape Differences Affect the Entire System

Koichiro Hayashi, Toshiki Yanagisawa, Ryo Kishida, and Kunio Ishikawa*

Department of Biomaterials, Faculty of Dental Science, Kyushu University, 3-1-1 Maidashi, Higashi-ku, Fukuoka, 812-8582, Japan

*Corresponding author.

E-mail address: khayashi@dent.kyushu-u.ac.jp

KEYWORDS. Honeycomb; Scaffold; Tissue engineering; Granule; Regenerative medicine; Bone.

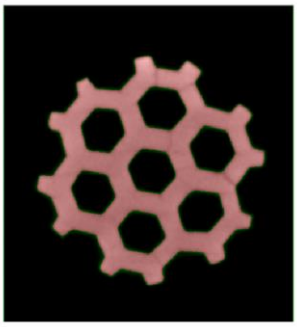

Figure S1. Example of the μ-CT images used for calculating macroporosity by image analysis. The whole scaffold was set as the region of interest (ROI). The strut region (red) was easily extracted from the whole scaffold. Conversely, the channel region was also easily extracted from the whole scaffold. Using the volumes of these regions, the macroporosity was calculated from Eq. 1 in the main text.

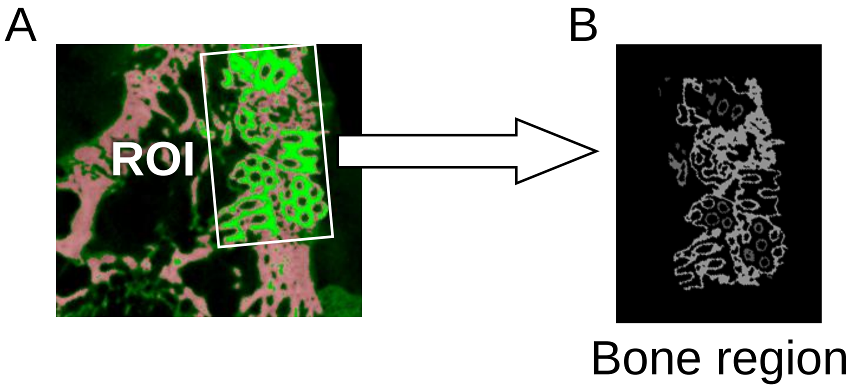

Figure S2. Example of the quantitative analysis approach of μ-CT data. (A) Extraction of the bone region of the ROI. In the ROI, vivid green indicates the remaining scaffold region and pink indicates the bone region. (B) Extracted bone region. Bone tissues formed in the intrascaffold channels and between scaffolds were clearly extracted.
